# Light and transmission electron microscopy of *Cepedea longa* (Opalinidae) from *Fejervarya limnocharis*

**DOI:** 10.1051/parasite/2017006

**Published:** 2017-02-01

**Authors:** Can Li, Xiao Jin, Ming Li, Guitang Wang, Hong Zou, Wenxiang Li, Shangong Wu

**Affiliations:** 1 Hubei Key Laboratory of Animal Nutrition and Feed Science, Wuhan Polytechnic University Wuhan 430023 PR China; 2 Key Laboratory of Aquaculture Disease Control, Ministry of Agriculture, Institute of Hydrobiology, Chinese Academy of Sciences Wuhan 430072 PR China

**Keywords:** *Cepedea longa*, *Fejervarya limnocharis*, Morphology, Opalinid, Ultrastructure

## Abstract

*Cepedea longa* Bezzenberger, 1904, collected from *Fejervarya limnocharis* (Amphibia, Anura, Ranidae) from Honghu Lake, Hubei Province, China in May–July 2016, is described at both light and transmission electron microscope levels. This is the first electron microscopic study of this species. *Cepedea longa* possesses a developed fibrillar skeletal system, composed of longitudinal fibrillar bands and transversal fibrils as well as numerous thin microfibrils dispersed in the endoplasm, which may play an important role in morphogenesis and offer some resilience to deformations of the cell. Longitudinal microfibrils are polarizing elements of kineties, bordering the somatic kineties on the left side and possibly responsible for kinetosome alignment. Two types of vesicles exist in the somatic cortex: globular endocytotic vesicles and flattened exocytotic vesicles. As to the nuclei of *C. longa*, a thick microfibrillar layer was observed to attach to the cytoplasmic face of the nuclear envelope. This fact suggests no necessary connection between the presence of this microfibrillar layer and the number of nuclei. In addition, some unknown tightly-packed microtubular structures in the nucleoplasm were observed for the first time in opalinids; neither their nature nor physiological significance is known. A detailed list of all reported *Cepedea* species is included.

## Abstract (fr)

*Cepedea longa* Bezzenberger, 1904, prélevé chez *Fejervarya limnocharis* (Amphibia, Anura, Ranidae) du lac Honghu, province du Hubei en mai-juillet 2016, est décrit au microscope photonique et au microscope électronique à transmission. Il s’agit de la première étude au microscope électronique de cette espèce. *Cepedea longa* possède un système squelettique fibrillaire développé, composé de bandes fibrillaires longitudinales et de fibrilles transversales ainsi que de nombreuses microfibrilles minces dispersées dans l’endoplasme, qui peuvent jouer un rôle important dans la morphogenèse et offrir une certaine résilience aux déformations de la cellule. Les microfibrilles longitudinales sont des éléments polarisants des kinéties, bordant les kinéties somatiques du côté gauche et éventuellement responsables de l’alignement kinétosomique. Deux types de vésicules existent dans le cortex somatique : des vésicules endocytotiques globulaires et des vésicules exocytotiques aplaties. Quant aux noyaux de *C. longa*, une couche microfibrillaire épaisse a été observée, qui se fixe à la face cytoplasmique de l’enveloppe nucléaire. Ce fait ne suggère aucun lien nécessaire entre la présence de cette couche microfibrillaire et le nombre de noyaux. De plus, on a observé pour la première fois chez les opalinidés des structures microtubulaires étroitement entassées dans le nucléoplasme, mais on ne connaît ni leur nature ni leur signification physiologique. Une liste détaillée de toutes les espèces de *Cepedea* est incluse.

## Introduction

Opalinids are commonly regarded as endocommensals in the guts of cold-blooded vertebrates, mostly amphibians, and have no known pathological effect on their hosts [13]. The studies on opalinids started in 1683 when Leeuwenhoek first discovered these mouthless protozoa [15]. Purkinje & Valentin introduced the genus *Opalina* Purkinje & Valentin, 1835 to include *Bursaria ranarum* Ehrenberg, 1832 [45]. Metcalf provided a clear definition of the genus *Opalina* and created three more genera *Protoopalina* Metcalf, 1918, *Cepedea* Metcalf, 1920 and *Zelleriella* Metcalf, 1920 [29, 30]*.* He assigned the “cylindrical binucleated species” to the genus *Protoopalina*, “flattened binucleated species” to the genus *Zelleriella*, “cylindrical multinucleated species” to the genus *Cepedea* and “flattened multinucleated species” to the genus *Opalina.* Earl added *Hegneriella* Earl, 1971 and *Bezzenbergeria* Earl, 1973, which are less widely accepted genera [17, 18]. Delvinquier et al. erected the fifth definite genus *Protozelleriella* Delvinquier et al. 1991 as a flattened binucleate species characterized by a peripheral hyaline area devoid of flagella and with a crenulate posterior margin [10].

As to the phylogenetic affinities, opalinids were for a long time regarded as “protociliates” [4, 29, 31]. Then the hypothesis of opalinid-ciliate affinity was abandoned with the better understanding of ciliate biology. As a result, the opalines were transferred from the ciliates and placed with amebae and flagellates either as an isolated taxon in the phylum Zooflagellata or were treated as a separate phylum: Opalinata [5, 6, 54]. Recent works based on detailed ultrastructural study and convincing phylogenetic analyses suggest that opalinids belong to heterokonts as a sister group to *Proteromonas* Kunstler, 1883 within the order Slopalinida, with two families, the Proteromonadidae and the Opalinidae [8, 26–28, 35, 41, 43–46].

*Cepedea* is a common genus of the family Opalinidae that inhabits anuran amphibians. It was created by Metcalf in 1920 when he assigned the “cylindrical multinucleated species” to this genus and placed the “flattened multinucleated species” into the genus *Opalina* [30]. Metcalf also considered that *Opalina* arose from *Cepedea* by flattening the body [32]. Mohr stated that “*Cepedea* is not a valid genus” because “flattened *Opalina* and cylindrical *Cepedea* have no discernible boundary” [38]. The application of protargol (silver proteinate) impregnation revealed the arrangement of the kineties, which stem from the falx [9–12, 21, 55]. On the basis of the arrangement of the falx relative to the anteroposterior axis of the body, Delvinquier & Patterson proposed a more recent definition of *Cepedea*: Multinucleated, with a short, broad, axial falx almost parallel to the anteroposterior axis of the cell; kineties cover the body evenly [7].

To date, many new species of *Cepedea* have been found from anuran amphibians (Table 1). *Cepedea longa* was first discovered and named by Bezzenberger in 1904 from the intestines of *Fejervarya limnocharis* (= *Rana limnocharis*) [3]. Thereafter, the redescription and some revisions of this species were given by Metcalf [31] and Nie [40], respectively. However, the morphological data were still incomplete since no transmission electron microscopic study had been carried out. The present study therefore attempts to supplement the morphology-based descriptions at both light and transmission electronic microscopic levels, aiming to contribute to the knowledge of this genus and to provide useful information for its taxonomy.

**Table 1.** The first discoveries of *Cepedea* spp. from anuran amphibians.

Year	Species	Author	Host	Locality	Reference
1860	*C. dimidiata*	Stein	*Rana esculenta*	Europe	Stein (1867) [47]
1904	*C. lanceolata*	Bezzenberger	*Rana esculenta*	Asia	Bezzenberger (1904) [3]
	*C. longa*	Bezzenberger	*Rana limnocharis*	Asia	Bezzenberger (1904) [3]
1922	*C. affinis*	Nazaretskaja	*Heterixalus madagascariensis*	Africa	Nazaretskaja (1922) [39]
1923	*C. baudinii*	Metcalf	*Hyla haudinii*	Guatemala; Cordova, Mexico	Metcalf (1923) [31]
	*C. borneonensis*	Metcalf	*Bufo jerboa*	Western Borneo	
	*C. buergeri*	Metcalf	*Polypedates buergeri*	Iga, Hondo, Japan	
	*C. buergeri sinensis*	Metcalf	*Bufo gargarizans*	Hong Kong, China,	
	*C. cantabrigesis*	Metcalf	*Rana cantabrigensis*	Manitoba, Canada; Alaska, USA	
	*C. dolichosoma*	Metcalf	*Bufo haematiticus*	Costa Rica	
	*C. floridensis*	Metcalf	*Scaphiopus alhus*	Key West, Florida	
	*C. formosae*	Metcalf	*Bufo melanostictus*	Hong Kong, Formosa, China	
	*C. fujiensis*	Metcalf	*Bufo formosus*	Fuji, Japan	
	*C. globosa*	Metcalf	*Phyllomedusa lemur*	Turrialba	
	*C. hispanica*	Metcalf	*Rana esculenta hispanica*	Alicante Province, Spain	
	*C. madagascariensis*	Metcalf	*Hyperolius marmoratus*	West Africa	
	*C. magna*	Metcalf	*Bufo latifrons*	Cameroon, West Africa	
	*C. mexicana*	Metcalf	*Rana pipiens*	Matamoros, Tamaulipas, Mexico	
	*C. minor*	Metcalf	*Alytes obstetricans*	Central France	
	*C. multiformis*	Metcalf	*Polypedates schleglii*	Yokohama, Japan	
	*C. obovoidea*	Metcalf	*Bufo lentiginosus*	Auburndale, Florida	
	*C. occidentalis*	Metcalf	*Rana chrysoprasina*	Nicaragua	
	*C. ophis*	Metcalf	*Rana tigerina*	Formosa, China; Billeton Island	
	*C. phrynomantidis*	Metcalf	*Phrynomantis bifasciata*	Tana, Africa	
	*C. pulchra*	Metcalf	*Kaloula pulchra Gray.*	Cochinchina	
	*C. pulchra japonica*	Metcalf	*Rana rugosa*	Nara, Yamoto Province, Japan	
	*C. pulchra javensis*	Metcalf	*Bufo melanostictus*	Buitenzorg, Java	
	*C. saharana*	Metcalf	*Rana esculenta ridibunda*	Biskra, Algeria	
	*C. segmentata*	Metcalf	*Polypedates leucomystax*	Cochinchina; Buitenzorg, Java	
	*C. seychellensis*	Metcalf	*Megalixalus seychellensis*	Mahé Island, Seychelles	
	*C. spinifera*	Metcalf	*Oxyglossus lima*	Buitenzorg, Java	
1923	*C. sudafricana*	Fantham	*Bufo regularis*	South Africa	Fantham (1923) [19]
1940	*C. lemuriae*	Metcalf	*Boophis rhodoscelis*	Madagascar	Metcalf (1940) [33]
1954	*C. africana*	Tuzet & Zuber-Vogeli	*Hyperolius concolor concolor*	Daloa, Ivory Coast	Tuzet & Zuber-Vogeli (1954) [49]
	*C. daloalensis*	Tuzet & Zuber-Vogeli	*Hemisus guineensis*	Daloa, Ivory Coast	
1965	*C. crispata*	Boisson	*Hyperolius viridiflavus*	Dakar, Senegal	Boisson (1965) [4]
1968	*C. boissoni*	Tuzet & Knoepffler	*Hyperolius viridiflavus*, *H. fusciventris*, *H. lamottei*	Lamto, Ndenou, Ivory Coast; Grassfield (Mt Nimba), Liberia	Tuzet & Knoepffler (1968) [48]
	*C. fusiformis*	Tuzet & Knoepffler	*Afrixalus doralis*	Lamto, Ivory Coast	
1993	*C. acuta*	Delvinquier et al.	*Tomopterna cryptotis*	Swaziland	Delvinquier et al. (1993) [11]
	*C. vanniekerkae*	Delvinquier et al.	*Tomopterna cryptotis*	South Africa	
1996	*C. couillardi*	Affa’a et al.	*Acanthixalus spinosus*	Cameroon Plateau, Yaoundé	Affa’a et al. (1996) [1]

## Materials and methods

The frogs *F. limnocharis* used for this study were captured from Honghu Lake (29°40′–29°58′ N; 113°12′–113°26′ E), Hubei Province, China in May–July 2016. We obtained the permits allowing us to capture and sacrifice these specimens. The frogs were transported alive to the laboratory for further examination. All frog samples were dissected as soon as possible. The recta were collected into Petri dishes and examined with the aid of Stemi SV6/AxioCam MRc5 (Zeiss, Oberkochen, Germany). The opalinids were collected with Pasteur micropipettes and washed twice in 0.65% saline solution.

For identification, specimens were smeared on coverslips and stained with ammoniacal silver carbonate [20] or silver nitrate [53]. For measurements, we used freshly killed specimens (in 5% formalin) with no coverslips mounted (except for the nucleus, which was measured in the ammoniacal silver stained slides). The specimens were observed, measured at 200× or 400× magnification and photographed using Axioplan 2 imaging and Axiophot 2 (Zeiss, Oberkochen, Germany). All measurements are in micrometers. Slides 2016W001-004 of silver nitrate stained specimens and 2016W005-010 of ammoniacal silver stained specimens have been deposited at the Institute of Hydrobiology, Chinese Academy of Sciences, Wuhan, China.

For transmission electron microscopy (TEM), specimens were fixed directly in 2.5% glutaraldehyde in 0.2 M phosphate-buffered saline (PBS, pH 7.4) for 2 h at 4 °C, then postfixed in 1% (v/v) osmium tetroxide in PBS for 2 h at 4 °C, followed by dehydration in a gradient acetone series and embedded in Araldite. Ultrathin sections were cut on a Leica Ultracut R ultramicrotome (Leica, Germany), stained with uranyl acetate and lead citrate before being observed in a JEM-1230 Transmission Electron Microscope (JEOL, Japan).

## Results

**Figure 1.**
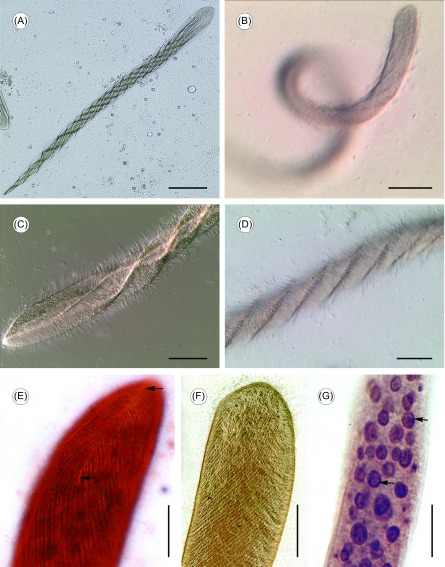
Light microscope images of *Cepedea longa.* (A) Overview of the living specimens, to show general form, greatly elongated and cylindrical, with the anterior extremity broader and the posterior end pointed. Scale bar = 100 μm. (B) Living specimens, to show *C. longa* thickly flagellated and often coils when swimming. Scale bar = 100 μm. (C)–(D) Living specimens, to show body surface twisting and giving a spiral appearance. Scale bar = 50 μm. (E) Specimens stained with ammoniacal silver, to show the falx (arrow) and somatic kineties branching off from each side. Scale bar = 25 μm. (F) Specimens stained with silver nitrate, to show somatic kineties follow a sigmoid course from anterior to posterior end of the cell. Scale bar = 25 μm. (G) Specimens stained with ammoniacal silver, to show the organism possessing a large amount of spherical or ellipsoidal nuclei (arrow). Scale bar = 25 μm.

Based on our survey, 76 (35.8%) of 212 examined *F. limnocharis* were found to be infected with *Cepedea longa*. Numerous opalinids were found mainly in the recta of frogs. The body is greatly elongated and cylindrical in form, slightly flattened and wedge-shaped at the anterior extremity, with the posterior end tapering or sharply pointed (Figs. 1A and 1C). Body length is 508.8–816.0 μm ( *n* = 20) and width 36.0–57.6 μm (; *n* = 20) *in vivo*. The animal is thickly flagellated and often coils when swimming (Fig. 1B), with its body surface twisting and giving a spiral appearance (Figs. 1C and 1D). The falx is quite short and thus difficult to observe, located at the margin of anterior extremity and parallel to the anteroposterior axis of the cell (Fig. 1E). All somatic kineties branch off from each side of the falx and follow a sigmoid course, numbering 64–87 (*n* = 8) in total (Figs. 1E and 1F). The organism possesses a large number of spherical or ellipsoidal nuclei (75–170; ; *n* = 20), with a diameter ranging from 4.5 μm to 10.4 μm (; *n* = 40) (Fig. 1G). Data for measurements related to morphometric characteristics are given in Table 2.

**Table 2.** Biometrical data (in μm) on *Cepedea longa* and comparison with former reports.

Host species	Locality	Parameter					Source of data
			BL	BW	Nnu	Dnu	
*F. limnocharis*	Diaocha Lake, Hubei Province, China		727.7	46.9	129.6	7.5	Present study
		M	748.8	48.0	132	7.8	
		Max	816.0	57.6	170	10.4	
		Min	508.8	36.0	75	4.5	
		SD	82.7	6.2	29.5	1.7	
		CV (%)	11.4	13.1	22.8	23.2	
		N	20	20	20	40	
*F. limnocharis*	Nanking, Jiangsu Province, China		1,162.0	42.5	–	5.7	Nie (1935) [40]
		Max	1,820.0	–	–	7.6	
*F. limnocharis*	Tokyo, Japan; Gillan, Formosa		1,000.0	75.0	–	–	Metcalf (1923) [31]
		Range	–	–	–	3.2–5.5	
*F. limnocharis*	Medak, India		680.0	52.0	–	–	Bezzenberger (1904) [3]
		Range	–	–	–	4.5–7.5	

Measurements in μm; , M = median, Max = maximum, Min = minimum, SD = standard deviation, CV = coefficient of variation, Nnu = number of nucleus, Dnu = diameter of nucleus.

**Figure 2.**
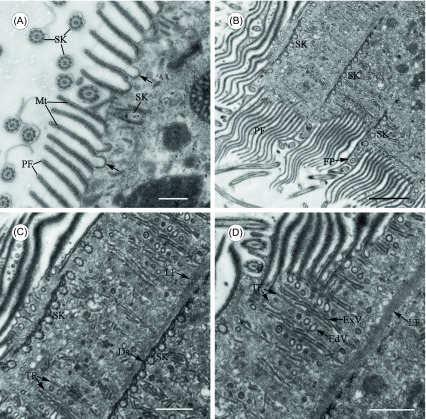
Transmission electron microscope images of *Cepedea longa*, to show fine structures of the somatic cortex. (A) Section tangent to cell surface, to show pellicular folds (PF) supported by ribbons of microtubules (Mt). Some coated vesicles are fused with the plasma membrane and seen as invaginations (arrow). SK = somatic kinetosomes. Scale bar = 5 μm. (B) Section passing parallel to cell surface, to show pellicular folds (PF) interposing between somatic kineties (SK). FP = flagellar pit. Scale bar = 20 μm. (C)–(D) Selected enlargement of Figure 2A, to show a developed fibrillar skeletal system in the somatic cortex. Longitudinal microfibrils (LF) border the somatic kineties (SK) joined to each other by desmoses (Ds) on the left side, with transversal fibrils (TF) running perpendicular to kinetal long axes and framing the ribs of the cortical vesicles: globular endocytotic vesicles (EdV) and elongated exocytotic vesicles (ExV). Scale bar = 10 μm.

**Figure 3.**
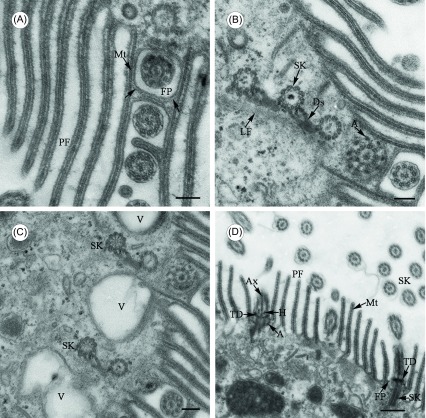
Transmission electron microscope images of *Cepedea longa*, to show fine structures of the somatic flagella. (A) Tangential section of a somatic kinety, to show fibrillar elements (arrow) between cortical microtubules (Mt) and around the membrane of each flagellar pit (FP). PF = pellicular folds. Scale bar = 2.5 μm. (B)–(C) Cross section through several kineties, to show somatic kinetosomes (SK) linked by desmoses (Ds) and sometimes interposed by vacuoles (V) just beneath the cell surface. A = kinetosomal arms. Scale bar = 2.5 μm. (D) Longitudinal section of kinetosomes, to show detailed fine structures. The axosome (Ax) is embedded in the proximal margin of transitional discs (TD), with curving arms (A) extending out and up. H = transitional helix, Mt = microtubules, SK = somatic kineties, PF = pellicular folds, FP = flagellar pit. Scale bar = 5 μm.

With a transmission electron microscope, pellicular folds can be seen clearly, which are supported by ribbons of microtubules (Figs. 2A, 2B and 3A). Coated vesicles often occur beneath the cortical folds, some of which are fused with the plasma membrane and seen as invaginations (Fig. 2A). Pellicular folds vary between kineties, with their numbers varying at different intervals (Fig. 2B). Microfibrillar bands run through the cortex. In fact, a developed fibrillar skeletal system exists – it is made up of longitudinal fibrillar bands and fine transversal fibrils (Figs. 2C and 2D). Longitudinal microfibrils border the somatic kineties on the left side, with transversal branches running perpendicular to kinetal long axes and framing the ribs of the cortical vesicles (Figs. 2C and 2D). There are two types of cortical vesicles: globular endocytotic (endocytic/pinocytic) vesicles and elongated exocytotic (exocytic/membrane “recycling”) vesicles. Endocytotic vesicles are often found in rows and alternate with these exocytotic vesicles (Fig. 2D).

The somatic flagella emerge in cylindrical pits, around which there is also some skeletal material (Figs. 2B, 3A and 3D). The somatic kinetosomes are linked by desmoses, which have characteristic periodicity (Fig. 3B). Vacuoles are sometimes found between somatic kineties just beneath the cortical surface (Fig. 3C). Interkinetosomal desmoses are always composed of two parts: the trifurcated left branch and the right branch extend as one fibril to finally contact the left posterior of the next anterior kinetosome (Figs. 3B and 3C). The projecting part of a flagellum has a conventional (9 + 2) axonemal structure (Figs. 3A–3C). At a level slightly above the bases of the cortical folds, there is an electron-dense helix around the central pair of microtubules (Fig. 3D). The axosome is embedded in the proximal margin of the transitional plate (Fig. 3D). Each peripheral group of microtubules in the kinetosome gives rise to a curving arm (Fig. 3B) which extends out and up to make contact with the plasma membrane (Fig. 3D).

**Figure 4.**
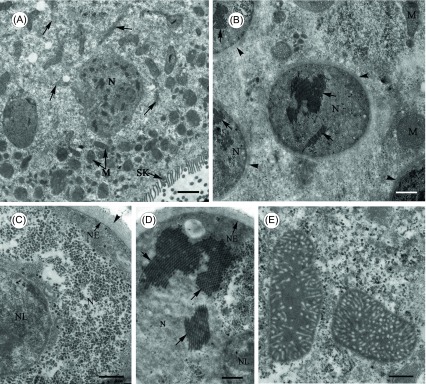
Transmission electron microscope images of *Cepedea longa*, to show fine structures of the nuclei and mitochondria within the endoplasm. (A) Cross section observed at low magnification, to show numerous thin bundles of microfilaments (arrow) dispersed in the endoplasm between nuclei (N) and mitochondria (M). SK = somatic kinetosomes. Scale bar = 20 μm. (B)–(D) Cross section of the nuclei (N), to show the nuclear envelope (NE) covered by a thick layer of microfibrils (arrowhead) and some unknown microtubular structures (arrow) in the nucleoplasm. NL = nucleolus. Scale bar in B = 10 μm, in C and D = 5 μm. (E) Thin section shows mitochondria having tubular cristae at periphery with an amorphic appearance. Scale bar = 5 μm.

Bundles of microfilaments can be observed crossing the endoplasm between nuclei and mitochondria (Fig. 4A). As a multinucleate opalinid, of course, *C. longa* has many nuclei in the cell (Fig. 4B). Each nucleus has one nucleolus in the nucleoplasm and a thick microfibrillar layer attached to the cytoplasmic face of the nuclear envelope (Figs. 4B and 4C). It is noteworthy that some unknown tightly-packed microtubular structures distribute in the nucleoplasm (Fig. 4D). Mitochondria have tubular cristae at their periphery and a relatively large volume of matrix with an amorphic appearance (Fig. 4E).

As to the falcular area, we failed to observe its ultrastructure because of its quite limited length, although we attempted many times to prepare thin sections. Hence, there is no description presented here.

## Discussion

As mentioned above, *C. longa* has been described from *F. limnocharis* by several authors. The average body size of opalinids examined in the present study (727.7 μm × 46.9 μm) bears the most resemblance to Bezzenberger’s type specimens (680.0 μm × 52.0 μm) [3], and is smaller than that recorded by Metcalf (1000.0 μm × 75.0 μm) [31] and Nie (1162.0 μm × 42.5 μm) [40]. The longest specimen of *C. longa* recorded by Nie even reaches 1820 μm in length [40]. These data reveal that *C. longa* varies greatly in body dimensions. They also suggest that body dimension is not a reliable taxonomic parameter for opalinids. According to the aforementioned studies, *C. longa* shows strict host specificity to *F. limnocharis* [3, 31, 40]. However, the host species has now been recognized as a cryptic species complex [14, 16]; thus, it is inappropriate to define *C. longa* as a host-specific endoparasite of *F. limnocharis,* since it shows at least some host variability. On the other hand, the body form and moving pattern of the living specimens, the arrangement of the falx and the nuclear features such as the number (mononucleated/binucleated/multinucleated), shape and position are most constant and important for specific identification [2, 7, 31].

The ultrastructural features of *C. longa* described herein closely resemble those of other opalinids: cortical folds supported by ribbons of microtubules, coated vesicles (pinocytotic) at the base of the folds, a developed cortical fibrillar system, delicate kinetosomal architectures, etc. The multiplication of cortical folds and coated vesicles found in *C. longa* is similar to that described in *C. dimidiata* Stein, 1860 [42], *C. sudafricana* Fantham, 1923 [37], *O. ranarum* Ehrenberg, 1832 [34, 43], *P. polykineta* Grim & Clements, 1996 [24] and *P. pomacantha* Grim et al., 2000 [25]. We think that the flattened exocytotic vesicles in rows under the cell surface may participate in the process of cell membrane reconstitution by which pinocytotic vesicles provide nutrients from the environment and then recycle back to the plasma membrane as the exocytotic, “membrane reconstruction” vesicles. This is a special adaptation strategy for these astomatous (no cytostome) opalinids.

According to our present study, *C. longa* possesses a developed fibrillar skeletal system, composed of longitudinal fibrillar bands and transversal fibrils as well as numerous thin microfibrils dispersed in the endoplasm. In fact, a network of microfibrils was also reported in some other opalines, such as *C. dimidiata* [42], *C. sudafricana* [37], *O. ranarum* [34, 43], *P. pseudonutti* Sandon, 1976 [36] and *P. pomacantha* [25]. These previous studies showed that the existence of a microfibrillar skeleton may not be a unique characteristic of the genus *Opalina* but possibly a common feature to all opalines. The microfibrillar networks also recall some ciliate skeletal components, in particular the ecto-endoplasmic boundary layer in some rumen ciliates [22, 23, 50–52]. As to their function, it is possible that they may play an important role in morphogenesis and offer some resilience to permanent deformations of the cell since the body is highly elastic and flexible. Moreover, these microfibrils, especially the longitudinal fibrillar bands, are polarizing elements of kineties and consequently may be responsible for kinetosome alignment.

With respect to the nuclei of *C. longa*, a thick microfibrillar layer was observed here to attach to the cytoplasmic face of the nuclear envelope. According to the study of Mignot and Affa’a [36], there is a similar fibrillar structure in *P. pseudonutti*, while the cytoplasmic face of the nuclear envelope is bare in *C. dimidiate*, *C. sudafricana* and *O. ranarum*. Hence, they stated that in different species of *Protoopalina* (having two nuclei per cell), the cytoplasmic face of the nuclear envelope is always covered with a microfibrillar layer, while in the multinucleate opalinids it was lacking [36]. However, our aforementioned observation in *C. longa* contradicts their hypothesis and suggests no necessary connection between this microfibrillar layer and number of nuclei. In addition, it is noteworthy that some unknown tightly-packed microtubules distributed in the nucleoplasm were observed in our present study. Hence, this is the first report of such microtubules in opalinids. Neither their nature nor physiological significance is known.
